# A chimeric dengue virus vaccine candidate delivered by high density microarray patches protects against infection in mice

**DOI:** 10.1038/s41541-021-00328-1

**Published:** 2021-05-07

**Authors:** Jovin J. Y. Choo, Laura J. Vet, Christopher L. D. McMillan, Jessica J. Harrison, Connor A. P. Scott, Alexandra C. I. Depelsenaire, Germain J. P. Fernando, Daniel Watterson, Roy A. Hall, Paul R. Young, Jody Hobson-Peters, David A. Muller

**Affiliations:** 1 Australian Infectious Diseases Research Centre, School of Chemistry and Molecular Biosciences, The University of Queensland, Brisbane, QLD Australia; 2 Vaxxas Pty Ltd, Translational Research Institute, Brisbane, QLD Australia

**Keywords:** Inactivated vaccines, Live attenuated vaccines, Dengue virus

## Abstract

Dengue viruses (DENV) cause an estimated 390 million infections globally. With no dengue-specific therapeutic treatment currently available, vaccination is the most promising strategy for its control. A wide range of DENV vaccines are in development, with one having already been licensed, albeit with limited distribution. We investigated the immunogenicity and protective efficacy of a chimeric virus vaccine candidate based on the insect-specific flavivirus, Binjari virus (BinJV), displaying the structural prM/E proteins of DENV (BinJ/DENV2-prME). In this study, we immunized AG129 mice with BinJ/DENV2-prME via a needle-free, high-density microarray patch (HD-MAP) delivery system. Immunization with a single, 1 µg dose of BinJ/DENV2-prME delivered via the HD-MAPs resulted in enhanced kinetics of neutralizing antibody induction when compared to needle delivery and complete protection against mortality upon virus challenge in the AG129 DENV mouse model.

## Introduction

Dengue is the most significant mosquito-borne viral disease throughout the world’s tropical zone^1,2^. Globally, an estimated 390 million cases of dengue virus (DENV) infections occur annually, resulting in a yearly approximated economic cost of US$6.9 billion, with 70% of the disease burden borne by Asian countries^1^. There are four serologically distinct serotypes, DENV 1–4, each of which are capable of causing a broad-spectrum disease in humans^3^. Patients infected with DENV can experience a range of clinical outcomes, depending on their age and immunocompetency. For those that develop symptoms, the clinical spectrum ranges from dengue fever, a self-limiting illness, to severe dengue haemorrhagic fever or potentially fatal dengue shock syndrome^4–6^. Upon primary infection, life-long immunity to that particular infecting serotype develops. However, immunity against the other three serotypes is short-lived. Consequently, cross-protection to heterologous serotypes following primary infection is limited, after which the risk of severe dengue disease increases when infected by a second virus serotype^3^. This increased susceptibility to severe disease following secondary infection with a heterologous virus serotype is explained by the phenomenon known as antibody-dependent enhancement^7,8^. It is postulated that the induction of cross-reactive antibodies bind to the secondary virus, facilitating virus entry through Fc-mediated endocytosis, resulting in increased viral replication and a dysregulated immune response leading to vascular leak and severe disease^9^.

With the possibility that secondary infection with a heterotypic virus serotype may lead to severe disease, the consensus is that a successful vaccine strategy requires an approach incorporating antigens of all four virus serotypes. Such a tetravalent vaccine would need to induce potent neutralizing antibodies against all four virus serotypes without priming for severe disease. Given the limitations of the current licensed vaccine, which has taken this approach^10,11^, alternative vaccine strategies are being actively pursued.

We have recently reported the development of a new chimeric virus system to produce flaviviral vaccine candidates using a newly discovered insect-specific flavivirus (ISF) named Binjari virus (BinJV)^12^. Exchange of the *prM/E* genes of BinJV with those of pathogenic flaviviruses results in chimeric virions that structurally mimic the pathogen and replicate to high titres in mosquito cell culture, but retain the insect-specific phenotype of the ISF, rendering them incapable of replicating in vertebrate cells^12^. The safety profile of these chimeric viruses, in addition to the authentic presentation of potent neutralizing antibody-eliciting quaternary epitopes, makes these chimeric viruses an excellent alternative vaccine candidate^12^. As such, the efficacy of the BinJV-based chimeras to protect against viral challenge in mouse models for Zika (ZIKV), West Nile (WNV) and yellow fever (YFV) viruses have already been established^12–14^. However, producing potent virus-neutralizing antibodies from vaccination with non-replicating vaccines can be challenging, and so we explored the use of the high-density microarray patch (HD-MAP) for vaccine delivery^15–17^.

The HD-MAP is a solid injection moulded 1 cm × 1 cm polymer high-density microprojection array containing 5000 projections of 250 µm in length onto which vaccine is dry-coated^18^. Using a spring-loaded applicator, the HD-MAP delivers the vaccine directly into the epidermal and upper dermal layers of the skin, which contain a high concentration of antigen-presenting cells (APCs)^19^. The dynamic application and delivery of vaccine antigen by the HD-MAP induces the co-localization of damage-associated molecular patterns and vaccine-related pathogen-associated molecular patterns. The co-localization of these signals with APCs are thought to be responsible for the enhanced immune responses regularly observed when vaccines are delivered via the HD-MAP^20,21^. Furthermore, the HD-MAP enables fractional dosing when compared to standard injection methods in mouse and rat models^15,19^.

Here we investigate the ability of HD-MAP delivery of the novel BinJ/DENV2-prME to induce a robust immune response and protect against virus challenge in a mouse model.

## Results

### Vaccine production and quantification

**Fig. 1:**
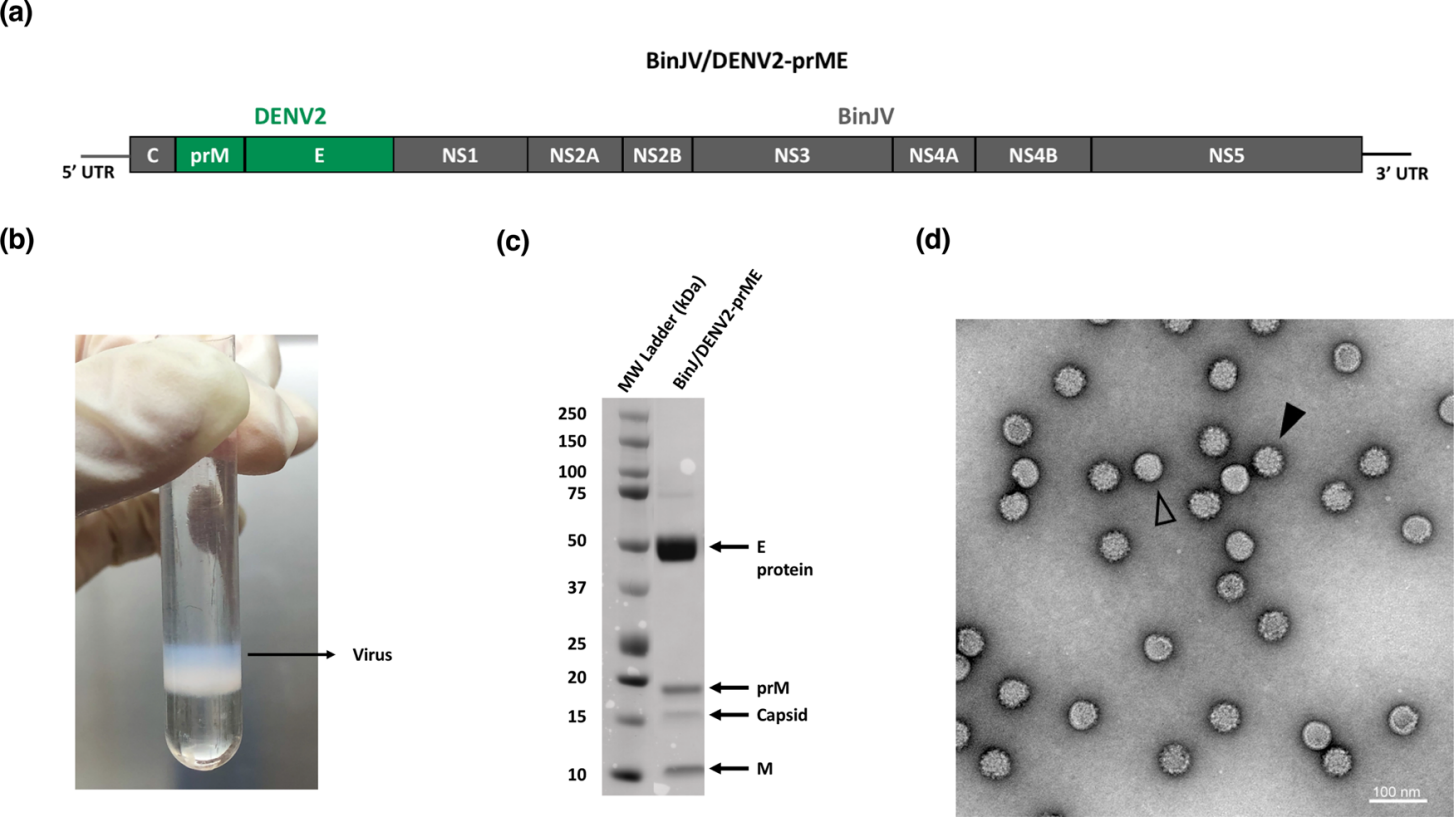
BinJ/DENV2-prME vaccine antigen production and analysis. **a** BinJ/DENV2-prME chimeric virus genome schematic with the *prM/E* genes of DENV2 indicated in green and BinJV capsid (C) and *NS* genes in grey. **b** BinJ/DENV2-prME was purified via a potassium tartrate gradient, sedementing as a wide opalescent blue band. **c** SDS-PAGE (4–12%) analysis of gradient-purified BinJ/DENV2-prME. Flavivirus structural proteins (pre-membrane (prM), C, membrane (M) and envelope (E) protein) are indicated. Gels were derived from the same experiment and processed in parallel. **d** Negative-stain TEM image of BinJ/DENV2-prME at ×30,000 magnification, highlighting representative mature (open arrowheads) and immature (closed arrowheads) viral particles.

The characterization of the BinJ/DENV2-prME chimera has been previously described^12^. In that study, chimeric virus replication kinetics in mosquito cells, antigenic authenticity and inability to replicate in vertebrate cells were confirmed^12^. In the current study, BinJ/DENV2-prME vaccine antigen (Fig. 1a) was prepared and purified via a potassium tartrate gradient, yielding a wide opalescent blue band (Fig. 1b). Sodium dodecyl sulfate-polyacrylamide gel electrophoresis (SDS-PAGE) analysis and total protein staining revealed a preparation comprising the DENV structural proteins, including the presence of prM, indicative of a heterogenous population of mature and immature particles (Fig. 1c). The presence of immature particles is typical for DENV cultures due to the relative inefficiency of the furin cleavage motif^22^. Transmission electron microscopy (TEM) analysis confirmed these observations (Fig. 1d). Using comparative densitometry, the concentration of virus was determined to be 4.67 mg/mL. The purified chimeric virus material was used for all subsequent immunization studies.

### Comparative immune responses to DENV2 VLP and BinJV/DENV2-prME in AG129 mice

**Fig. 2:**
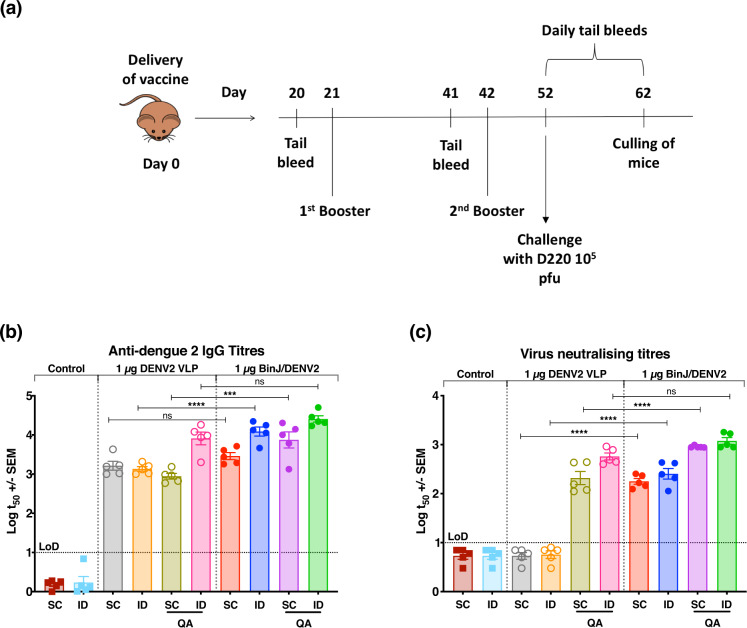
Evaluation of BinJ/DENV2-prME and DENV2 VLP vaccines in female AG129 mice. **a** Experiment time course. Female AG129 mice (*n* = 5) were vaccinated via ID or SC injection with 1 µg of BinJ/DENV2-prME or DENV2 VLP, with or without 3 µg of QA. Booster doses were given on days 21 and 42, and tail bleeds were taken on days 20, 41 and 51 after the first vaccination. Mice were challenged 10 days post final vaccination and daily tail bleeds were taken for 10 days to monitor survival, viremia and circulating NS1 levels. **b** The IgG response was determined for sera taken 10 days after the final vaccination and mid-point antibody titres (t50) plotted. **c** Neutralizing antibody titres 10 days post final vaccination expressed as PRNT_50_ against DENV ET00. Note: each symbol represents a single mouse. Lines indicate mean antibody titres with bars showing ±SEM. ****p ≤ 0.0001, ***p ≤ 0.0003, **p ≤ 0.002, *p ≤ 0.03 assessed by one-way analysis of variance (ANOVA, α-level 0.05).

As the BinJ/DENV2-prME chimera is unable to replicate in vertebrate cells, it may be viewed as being similar to an inactivated whole virus vaccine or a virus-like particle (VLP)-like vaccine. So, to evaluate the BinJ/DENV2-prME vaccine candidate, a comparative study was performed in AG129 mice to assess the performance of the chimeric BinJ/DENV2-prME antigen alongside a commercially available recombinant VLP-based antigen comprising DENV2 prM/E produced transiently in HEK293 human cells^23^. Female AG129 mice were vaccinated three times, 21 days apart, with 1 µg doses of either BinJ/DENV2-prME or the DENV2 VLP, delivered by intradermal (ID) and subcutaneous (SC) injection with and without the adjuvant Quil-A (QA) (Fig. 2a). Anti-DENV IgG responses were detected in all vaccinated mice after administering three doses, regardless of the antigen or route (Fig. 2c). When administrated via SC, significant differences in the anti-DENV IgG response were observed between the adjuvanted BinJ/DENV2-prME and DENV2 VLP groups (Fig. 2b, *p* ≤ 0.0001) but not the unadjuvanted counterpart (Fig. 2b, *p* = 0.9033). However, the opposite was discovered for the ID vaccinated groups with significantly higher IgG titres observed between BinJ/DENV2-prME and DENV2 VLP without QA (Fig. 2b, *p* < 0.0001) but not their adjuvanted counterparts (Fig. 2b, *p* = 0.1351). To examine the virus-neutralizing potential of the induced IgG, plaque reduction neutralization tests (PRNTs) were also performed (Fig. 2c). Neutralizing antibodies were detected for all vaccinated groups, except for those that received the DENV2 VLP without the QA adjuvant, indicating that a 1 µg dose of DENV VLP is not sufficient to induce a detectable neutralizing antibody response. Significantly higher neutralizing antibody titres were observed for mice vaccinated with BinJ/DENV2-prME via either ID or SC routes when compared to those receiving the DENV2 VLP (Fig. 2c, *p* < 0.0001). When BinJ/DENV2-prME was administered with the adjuvant QA, higher neutralizing antibody titres were induced via only the SC route (Fig. 2c, *p* < 0.0001).

### BinJ/DENV2-prME provides protection against DENV challenge

**Fig. 3:**
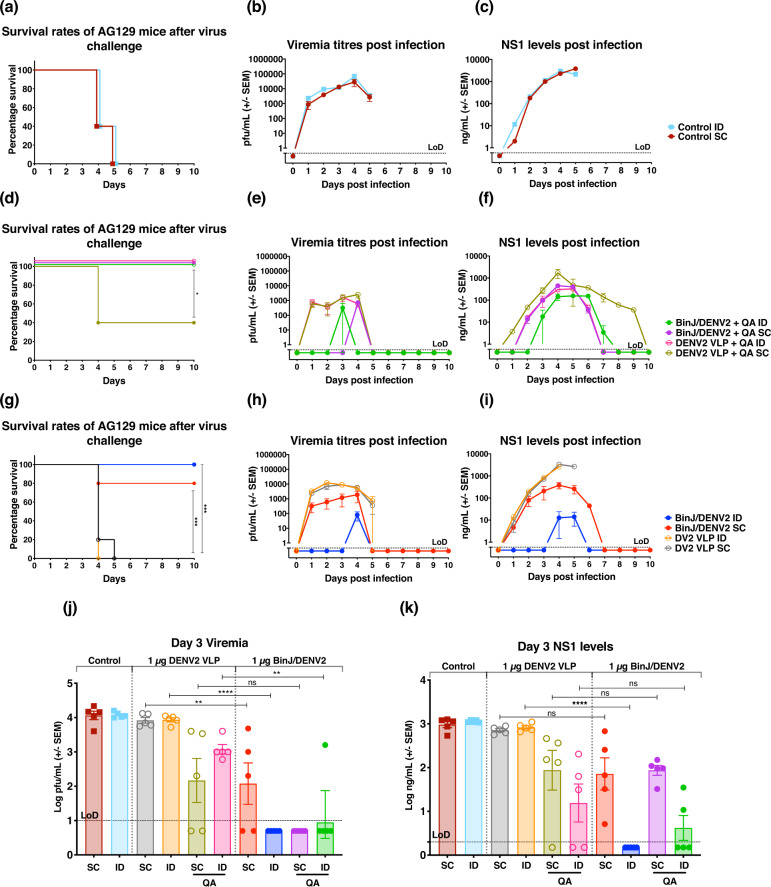
Survival, viremia and NS1 secretion in mice following immunization and challenge. **a**, **d**, **g** Survival rates of mice over 10 days post challenge. **b**, **e**, **h** Mean viremia was determined by plaque assay on Vero cells. Limit of detection was 50 pfu/mL. **c**, **f**, **i** Serum levels of NS1 were determined using quantitative capture ELISA. To demonstrate the significant differences between **j** viremia and **k** NS1 levels, data from day 3 post virus challenge were plotted. Each symbol represents a single mouse. Lines indicate mean antibody titres with bars showing ±SEM. ****p ≤ 0.0001, ***p ≤ 0.0003, **p ≤ 0.002, *p ≤ 0.03 assessed by one-way analysis of variance (ANOVA, α-level 0.05).

Given the induction of a potent virus-neutralizing antibody, we examined the capacity of BinJ/DENV2-prME to elicit protection from challenge with a mouse-adapted virus isolate, DENV2 D220. Ten days after the third vaccination, mice were challenged with the experimentally determined dose of 10^5^ plaque forming units (pfu) mouse-adapted DENV2, D220. In addition to clinical scoring and weight loss (Supplementary Fig. 1), the kinetics of viremia and non-structural 1 (NS1) secretion were also determined. Non-vaccinated control mice showed severe clinical signs (Supplementary Fig. 1) accompanied with a rapid increase in viral load and NS1 levels within the first 5 days, at which point the mice were culled based on disease morbidity scoring (Fig. 3a–c).

All groups vaccinated with adjuvant afforded complete protection, except for the DENV2 VLP + QA SC group, which resulted in 40% survival (Fig. 3d). The survival rate coincided with the morbidity scores of the DENV2 VLP + QA SC group peaking at day 4 of the challenge period, when mice were culled (Supplementary Fig. 1b). Data obtained from day 3 post virus challenge showed significant reduction in viremia and NS1 levels in mice receiving unadjuvanted BinJ/DENV2-prME, except for the SC vaccinated mice, which had no significant differences in NS1 level compared to their VLP counterparts (Fig. 3j, k). Although there was no significant difference between peak viral load and NS1 levels (as assessed at 3 days post challenge) in mice receiving BinJ/DENV2-prME + QA via SC, significantly lower viral loads were observed for the ID vaccinated groups (Fig. 3j, k). Overall, delayed kinetics was observed for both these biomarkers in mice receiving BinJ/DENV2-prME + QA as compared to VLP groups (Fig. 3e, f, j, k).

Mice vaccinated with DENV2 VLPs without adjuvant, followed a similar progression of disease, viral load and NS1 levels to the control mice, and were humanely culled on days 4 and 5 of the challenge (Fig. 3g–i). In contrast, vaccination with BinJ/DENV2-prME in the absence of adjuvant resulted in 100% and 80% survival for those mice injected ID and SC, respectively (Fig. 3g). Furthermore, ID immunization with BinJ/DENV2-prME without adjuvant resulted in significantly lower peak viral load and NS1 levels as compared to their VLP counterparts (Fig. 3h, I, j, k).

### Vaccine coating and delivery via HD-MAPs

**Fig. 4:**
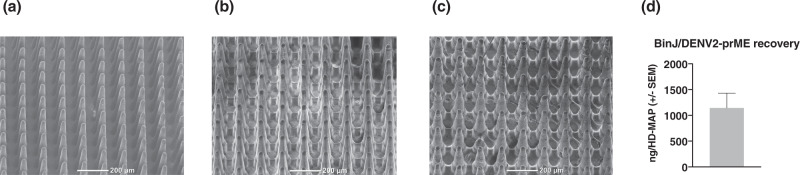
SEM images and delivery efficiency of BinJV/DENV2-prME-coated HD-MAP. SEM images of **a** an uncoated HD-MAP, **b** HD-MAP coated with BinJ/DENV2-prME and **c** HD-MAP post application, showing the removal of vaccine from the tips of the projections. **d** Vaccine dosage delivered by HD-MAPs were evaluated prior to immunization. Subtractive ELISA using antibodies against the E protein of DENV was performed to determine dose delivered. The bar graph represents the mean of BinJ/DENV2-prME (*n* = 5) delivered by HD-MAPs with error bars indicating SEM.

To build on the success of ID vaccination with BinJ/DENV2-prME, we investigated the utility of the HD-MAP delivery platform to further enhance immune responses. To deliver a known quantity/desired dose of vaccine, we performed coating and delivery optimization of BinJ/DENV2-prME by HD-MAP. HD-MAPs were coated with BinJ/DENV2-prME formulated with previously defined excipients, 0.75% methylcellulose and 0.75% trehalose. Uncoated (Fig. 4a) and coated HD-MAPs were imaged by scanning electron microscopy (SEM) to observe coating uniformity before (Fig. 4b) and after coating removal following HD-MAP application to the mouse flank (Fig. 4c). HD-MAP coating with the vaccine formulation revealed smooth and even distribution on the microprojections (Fig. 4b). Removal of vaccine was observed at the tip of the microprojections, confirming that the vaccine had been delivered into the mouse skin (Fig. 4c). To quantify the amount of delivered vaccine, a subtractive enzyme-linked immunosorbent assay (ELISA) was performed, measuring the amount of vaccine remaining on the microprojections following HD-MAP application. The amount of vaccine delivered was calculated to be 1.1 µg per HD-MAP as shown in Fig. 4d. After successful coating and determination of delivered vaccine, we proceeded to evaluate HD-MAP delivery of BinJV/DENV2-prME in the AG129 dengue mouse model.

### Immune response following HD-MAP vaccination

**Fig. 5:**
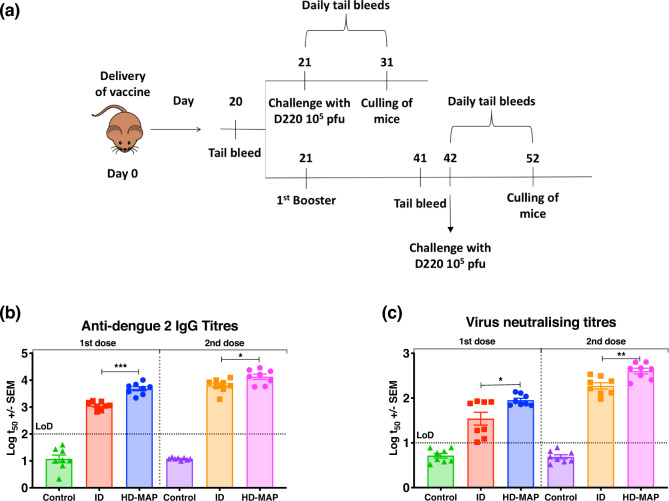
Evaluation of BinJ/DENV2-prME and DENV2 VLP vaccines in female AG129 mice. **a** Experimental time-course and vaccination regimen. Female AG129 mice (*n* = 8) were vaccinated through ID or HD-MAP with either 1 µg of BinJ/DENV2-prME or saline (control). The first group was challenged with 10^5^ pfu D220 after a single dose, whereas the second group was given a booster dose and challenged 21 days later. Tail bleeds were taken 20 days post first immunization for the single dose and day 41 for the two-dose groups. Following virus challenge, daily tail bleeds were taken for 10 days to monitor survival, viremia and NS1 levels. **b** The IgG response was determined for sera taken 10 days after the final vaccination and mid-point antibody titres (t50) plotted. **c** Neutralizing antibody titres 10 days post final vaccination expressed as PRNT_50_ against DENV2 ET00. Note: each symbol represents a single mouse. Lines indicates mean antibody titres with bars showing ±SEM. ****p ≤ 0.0001, ***p ≤ 0.0003, **p ≤ 0.002, *p ≤ 0.03 assessed by one-way analysis of variance (ANOVA, α-level 0.05).

Following the successful demonstration of protection from non-adjuvanted needle-based ID immunization, we evaluated protective efficacy of BinJ/DENV2-prME delivered by the HD-MAP in a single- or two-dose regimen. AG129 mice were immunized with 1 µg BinJ/DENV2-prME via ID injection or HD-MAP receiving one or two doses 21 days apart (Fig. 5a). Mice given a single dose of vaccine were challenged with 10^5^ pfu of D220 21 days post primary immunization, whereas those groups receiving a second dose were challenged 21 days after the second dose (Fig. 5a). Before each vaccination and virus challenge, blood samples were collected and the serum was analysed for virus-specific IgG and neutralizing antibody (Fig. 5a). Mice vaccinated via the HD-MAP had significantly higher anti-dengue IgG titres after a single dose compared to the ID vaccinated mice (Fig. 5b, *p* = 0.0005). An increase in IgG levels was observed after the second dose for both ID and HD-MAP groups, with higher antibody titres induced in the HD-MAP-vaccinated group (Fig. 5b, *p* = 0.01). To confirm the anti-DENV IgG from vaccinated animals was functional, we next investigated the ability of these antibodies to neutralize wild-type dengue (DENV2 ET200). Neutralizing antibody titres reflected vaccine-specific IgG responses with mice vaccinated with a single dose via the HD-MAP having significantly higher neutralizing antibody titres as compared to those vaccinated via ID (Fig. 5c, *p* = 0.01)).

The HD-MAP deposits vaccines directly to the APCs in the skin and it is known that DENV entry to dendritic cells is mediated by DC-SIGN^24^. To investigate whether the enhanced immune responses to BinJ/DENV2-prME were due to limited RNA replication or de novo translation, DC-SIGN-positive cell lines K-562 and A549 were selected for analysis along with control C6/36 cells. Infected cells at 6 and 24 h were stained for double-stranded RNA (dsRNA) and NS1 (BinJV-specific and pan-flavivirus). Wild-type DENV2 infection resulted in the detection of dsRNA and limited NS1 in all cell lines tested (Supplementary Fig. 2). As previously observed by Hobson et al.^12^, the chimeric BinJ/DNV2-prME was able to establish infection in C6/36 cells with both dsRNA and NS1 production (Supplementary Fig. 2a). However, the detection of dsRNA and NS1 was absent from the mammalian cell lines when infected with BinJ/DENV2-prME (Supplementary Fig. 2b, c).

### DENV challenge of HD-MAP-vaccinated AG129 mice

**Fig. 6:**
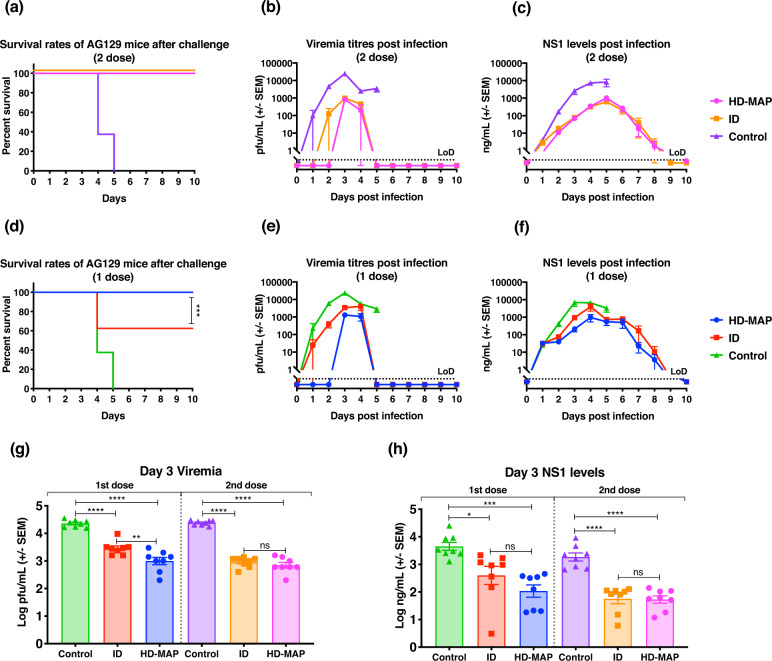
Survival, viremia and NS1 secretion following BinJ/DENV2-prME immunization. **a**, **d** Survival rates of mice over 10 days post challenge. **b**, **e** Mean viremias were determined by plaque assay on Vero cells. Limit of detection was 50 pfu/mL. **c**, **f** Serum levels of NS1 were determined using quantitative capture ELISA. To demonstrate the significant differences between **g** viremia and **h** NS1 levels, data from day 3 post virus challenge were plotted. Each symbol represents a single mouse. Lines indicates mean antibody titres with bars showing ±SEM. ****p ≤ 0.0001, ***p ≤ 0.0003, **p ≤ 0.002, *p ≤ 0.03 assessed by one-way analysis of variance (ANOVA, α-level 0.05).

To evaluate the protective efficacy of one- or two-dose vaccination with BinJ/DENV2-prME delivered by the HD-MAP and ID injection, we challenged vaccinated mice with 10^5^ pfu of D220. Mice were monitored for clinical scores (including animal weight, Supplementary Fig. 3), viremia, NS1 levels and survival for 10 days following challenge (Fig. 6). Control mice rapidly deteriorated, exhibiting disease symptoms (Supplementary Fig. 3), which were concomitant with rapidly increasing viral load and NS1 levels with all mice being culled on days 4 and 5 (Fig. 6a–c). Following two doses of BinJ/DENV2-prME via HD-MAP or ID injection, viral load and NS1 levels were significantly reduced as compared to the control mice (Fig. 6b, c, g, h). This reduction in NS1 and free circulating virus coincided with minimal disease symptoms and complete protection from virus challenge (Fig. 6a and Supplementary Fig. 2b).

Following the success of two-dose vaccination, we investigated the protective efficacy of a single-dose vaccination regimen. The control group showed the same magnitude and kinetics of viral load, NS1 levels and disease progression/survival as described above (Fig. 6d–f and Supplementary Fig. 2). The HD-MAP-immunized group showed minimal signs of disease on day 4 and 5 with 100% of mice surviving the viral challenge (Supplementary Fig. 2b and Fig. 6d, respectively). The ID vaccinated group showed similar morbidity scores throughout the monitoring period, although peaking higher on day 4 when compared to the HD-MAP group (Supplementary Fig. 2b). This difference reflects the partial protection of 62.5% observed in the ID vaccinated group (Fig. 6d). Infection biomarkers, viral load and NS1 levels were significantly reduced for both ID and HD-MAP groups when compared to the control group (Fig. 6b, c, g, h). Comparing the two delivery methods, mice receiving the vaccine via HD-MAPs had significantly lower viral load with free circulating virus not detected until day 3 post infection (Fig. 6b, g). In contrast, mice vaccinated by ID injection showed a rapid increase in viral load following infection resulting in partial protection.

## Discussion

The development and licensure of an effective dengue vaccine has been a priority for more than 50 years^25^. Here we describe the application of an ISF platform technology to the development of a BinJ/DENV2 chimeric virus as a dengue vaccine candidate. We further showed that vaccinated mice induced potent neutralizing antibodies against DENV, and that by combining the BinJ/DENV2-prME antigen with the HD-MAP vaccine delivery platform, complete protection from virus challenge of AG129 mice could be elicited using a single unadjuvanted dose of 1 µg of BinJ/DENV2-prME.

Although the licensure of Dengvaxia in 2016 was a major step forward in dengue vaccine development, limitations around its use because of safety concerns has ensured that the development of alternative vaccine strategies has remained a priority^26–28^. The two most advanced vaccine candidates currently in phase III clinical trials are DENVax by Takeda Vaccines (live attenuated chimeric virus with DENV2 backbone) and TV003/TV005 by US National Institute of Health (live attenuated Δ30 viruses)^29,30^. Subunit vaccine approaches employing the DENV E protein have also been explored, with their safety profile providing some advantages over live attenuated vaccines^15,31^.

There is a growing body of evidence that antibodies targeting quarternary epitopes, such as the dimer interface of the E protein, are important for virus neutralization. Although VLPs and inactivated whole virus vaccines have shown promise, it has been previously found that these vaccine candidates are not structurally identical to their infectious flavivirus counterparts^12–14^. These differences have been suggested to be due to presentation of the E dimers on the surface of the VLPs or through the chemical inactivation process of the whole virus vaccines^12–14^. However, the BinJV chimeras are structurally and antigenically identical to their infectious flavivirus counterparts^12,23^. In addition, although DENV VLP vaccine candidates have been shown to require administration over multiple doses along with an adjuvant^12–14^, we show in this study that a single unadjuvanted dose of BinJ/DENV2-prME can elicit solid protection in a mouse model.

Our group has previously shown that BinJ/vertebrate-infecting flavirius (VIF)-prME antigen are highly immunogenic in mice, using vaccine candidates displaying the prM/E for WNV, YFV and ZIKV^12–14,32^. Indeed, consistent with the BinJ/DENV2-prME vaccine candidate examined here, the analogous chimeras for ZIKV and WNV afforded complete protection in their respective mouse models using a 1 µg dose and without the use of adjuvant. Although immunization via ID with adjuvanted BinJ/DENV2-prME elicited potent IgG and neutralizing antibody titres, providing complete protection upon virus challenge, higher viral loads and NS1 levels were noted in this immunized group when compared to their unadjuvanted counterparts. By contrast, mice vaccinated via ID injections with BinJ/DENV2-prME without adjuvant achieved complete protection, suggesting that the addition of QA may not be necessary.

Vaccine administration by the ID route targets the dermis and epidermis of the skin, a location rich in APCs, and elicits more enhanced immune responses than SC or intramuscular injection^33^. However, it is also technically challenging to successfully administer an ID injection and requires a trained personnel^33^. To overcome this limitation, there has been a growing interest in alternative vaccine delivery platforms such as microneedles/microarray patches, which have shown varying degrees of efficacy in mouse models^19,34–36^. Using HD-MAPs, our group has previously shown the induction of enhanced immune responses with significant dose sparing^15–17,19^. Taking advantage of the dose reduction potential of the HD-MAP, we evaluated the protective efficacy of delivering BinJ/DENV2-prME to the skin via this approach. Our findings show that all mice receiving a single dose of BinJ/DENV2-prME delivered by HD-MAP had significantly reduced levels of viremia and NS1 when compared to control mice following virus challenge, and were fully protected (Fig. 5)^15^. Although these results are promising, one of the limitations of our study is the focus on a single serotype of DENV. As such, no comment can be made to the short-term vs. long-term protective efficacy of antibodies produced by the vaccination. Likewise, further investigation with regards to cross-reactivity with the other DENV serotypes or flaviviruses is warrented.

This is the primary study to demonstrate the effective delivery of a novel chimeric virus vaccine candidate, BinJ/DENV2-prME, by the HD-MAP. The targeted delivery of a single dose of the vaccine to the skin raised potent immune responses that afforded protection to DENV challenge in the AG129 mouse model. With the success of a single dose of BinJ/DENV2-prME delivered by HD-MAP, the production and evaluation of the remaining three dengue serotypes are currently under investigation. In addition to the successful pre-clinical studies using these chimeras for WNV, YFV and ZIKV, the efficacy of constructs for other flaviviral diseases are also being explored. Coupled with targeted skin delivery, these chimeric viruses offer a promising new vaccine platform for the future development of a wide range of flavivirus vaccines.

## Methods

### Animal ethics

Animal experiments were approved by the University of Queensland animal ethics committee (AEC No: SCMB/AIBN/150/16/NHMRC) and were performed in accordance with National Health and Medical Research Council guidelines. Animals were housed under specific pathogen-free conditions in the University of Queensland Biological Resources animal housing facility at the Australian Institute for Bioengineering and Nanotechnology.

### Cell lines

Vero cells (African green monkey kidney) were maintained in OPTI-MEM (Gibco) cell culture medium, containing 3% fetal bovine serum (FBS, Bovogen) at 37 °C with 5% CO_2_. C6/36 (*Aedes albopictus*) cells were grown in glutamate-free Roswell Memorial Park Institute-1640 (RPMI, Gibco) medium, supplemented with 10% FBS, 15 mM HEPES (Gibco), 1% GlutaMAX^TM^ (Gibco), 100 U/mL penicillin (Gibco) and 100 μg/mL streptomycin (Gibco) at 28 °C. K-562 cells (human bone marrow) were maintained in RPMI-1640, supplemented with 10% FBS, 100 U/mL penicillin (Gibco) and 100 μg/mL streptomycin (Gibco) at 37 °C with 5% CO_2_. A549 cells (human lung epithelial) were maintained in Dubecco’s modified Eagle medium (Gibco), supplemented with 10% FBS, 100 U/mL penicillin (Gibco) and 100 μg/mL streptomycin (Gibco) at 37 °C with 5% CO_2_.

### BinJ/DENV2-prME chimeric virus propagation and purification

The generation and production of BinJV/VIF-prME chimeras were previously described^12^. C6/36 cells at 80% confluency were infected with BinJ/DENV2-prME at a multiplicity of infection (MOI) of 0.01. Supernatant was collected at days 3, 5 and 7 post infection. The virus culture supernatant was clarified via centrifugation at 3000 × *g* for 15 min at 4 °C, filtered through a 0.22 µM filter (Millipore) and stored at 4 °C until purification. After each collection, cells were replenished with fresh RPMI media containing 2% FBS. The BinJ/DENV2-prME virions were precipitated via addition of polyethylene glycol 8000 (Sigma Aldrich) to a final concentration of 8% and slow stirring overnight at 4 °C. The virus was pelleted at 12,000 × *g* for 1 h at 4 °C, followed by ultracentrifugation through a sucrose cushion and potassium tartrate gradient as described by Warrilow et al.^37^. Purified virus was collected and buffer-exchanged into phosphate-buffered saline (PBS) using a 30 kDa Amicon filter (Millipore) and stored at 4 °C.

### SDS-PAGE analysis of purified virus

Purified viruses were run on a 4–12% Bis-Tris gel to confirm production, purity and molecular weight of viral protein (Supplementary Fig. 4). Samples were prepared with 4× SDS buffer and boiled for 5 min before SDS-PAGE separation. Gels were run at 150 V for 1 h. Gels were stained with GelCode Blue Stain (Thermo Fisher) according to the manufacturer’s instructions. Quantification of viral protein was performed as mentioned by Vet et al.^13^.

### Negative-stain TEM

BinJ/DENV2-prME particles were purified as described above. Purified virions were dried onto a freshly glow-discharged 200-mesh square carbon-coated copper grid (Proscitech). Grids were then stained with 2% uranyl acetate and imaged by TEM on a Hitachi HT 7700 at 100 kV.

### HD-MAPs coating and application

HD-MAPs (1 cm^2^, 5000 projections/cm^2^ at 250 µM in length) were kindly provided by Vaxxas Pty Ltd, Brisbane, Australia. HD-MAPs were plasma treated with oxygen for 3 min at Australian National Fabrication Facility before vaccine coating. Coating solution made up for five HD-MAP consisted of 19.7 µL of 4% methylcellulose, 15.75 µL of 5% trehalose and 5.35 µL of 4.67 mg/mL purified BinJ/DENV2-prME in low-salt buffer (20 mM Tris and 75 mM NaCl pH 7.4). The coating solution (21 µL) was added onto each patch and dry-coated on to the microprojections under a nitrogen jet stream at 15 m/s for 250 s, then 50 m/s for 90 s. Vaccine-coated HD-MAPs were applied to the flank of the mice at a velocity of 20 m/s using an applicator.

### Delivery efficiency

A subtractive DENV Envelope (E) protein ELISA was performed to determine the dose delivered to the flank by HD-MAPs. Briefly, 50 µL of 2 µg/mL of DENV antibody humanized C8^38^ in bicarbonate buffer (0.05 M carbonate–bicarbonate buffer pH 9.6, Sigma Aldrich) was coated on ELISA plates (Nunc Maxisorb, Thermo Fisher) overnight at 4 °C. Plates were blocked with blocking buffer (KPL, SeraCare) for 1 h at room temperature. To generate a standard curve, 1 µg/mL of BinJ/DENV2 was added and titrated via doubling dilutions across the plate. Remaining vaccines were eluted off coated, patched HD-MAPs and coated, unpatched HD-MAPs by pipetting blocking buffer onto all four corners and the middle of the HD-MAP five times. The eluted vaccines (50 µL/well) were then added to the ELISA plate and incubated at 37 °C for 1 h alongside the BinJ/DENV2-prME standard curve. Plates were then washed 3× with PBS–0.05% Tween-20 (PBS-T). DENV antibody humanized 513^39^, conjugated with horse-radish peroxidase (HRP, Abcam) according to the manufacturer’s instructions, was added and incubated at 37 °C for 1 h. Plates were then washed 6× with PBS-T and 50 µL/well of Tetramethylbenzidine-w (TMBW, Biofx) was added. Relative absorbance was read at 450 nm on a plate reader. Dose delivered was determined by measuring the difference between the amount of vaccine recovered from coated HD-MAPs, pre and post application.

### Scanning electron microscopy of HD-MAPs

HD-MAPs were imaged by SEM using a JEOL JCM-500 Neoscope with samples tilted at a 45° angle. Imaging of the HD-MAPs was performed as previously described^20^. All images were taken at high resolution at 10 kV.

### Immunofluorescence assays

Ninety-six-well black optical plates (Thermo Fisher) were treated with Poly-l-Lysine (Thermo Fisher) for 30 min at 37 °C. Plates were washed 2× with sterile PBS and left to dry overnight. A549 cells were seeded at 1 × 10^4^ cells/well and K-562 cells were seeded at 3 × 10^4^ cells/well overnight at 37 °C. C6/36 cells were seeded at 3 × 10^4^ cells/well overnight at 28 °C. Cells were infected with BinJ/DENV2-prME or DENV2 ET00 at an MOI of 1 or were mock infected and incubated at room temperature with rocking for 1 h. The virus inoculum was removed and cells were washed 3× with sterile PBS. Appropriate growth media for each cell line was added and incubated for 6 and 24 h post infection at 28 °C or 37 °C for the indicated mammalian and insect cell lines. Cells were fixed with 4% paraformaldehyde in PBS with 0.1% Triton X-100. Fixed plates were blocked in 1% bovine serum albumin (BSA)/PBS for 1 h at room temperature before adding 50 µL of mouse anti-3G1^40^ (1 : 3), mouse/human anti-4G4^41^ (1 : 10; 1 : 500), mouse anti-7G10^42^ (1 : 5) or mouse anti-DC-SIGN (1 : 500) mAb diluted in 1% BSA/PBS/0.1% Tween-20 to the respective wells. Plates were incubated for 1 h at 37 °C and then washed 4× with PBS-T. Fifty microlitres of secondary antibody Goat anti-mouse Alexa Fluor 488 or Goat anti-human Alexa Fluor 647 diluted 1 : 5000 were added into the respective wells and incubated at 37 °C for an hour. Plates were then washed 6× with PBS-T. Nuclear staining was performed with Hoechst 33342 (Thermo Fisher) 1 : 10,000 during the final wash with shaking for 5 min. One hundred microlitres of PBS were added into each well and plates were stored at 4 °C until imaging on a confocal microscope (Ziess 710).

### Immunization of AG129 mice with vaccine candidates: DENV2 VLPs and BinJ/DENV2-prME comparative study

Female AG129 mice (6–8 weeks old) were divided into ten groups of five mice each: immunization by ID or SC injections with BinJ/DENV2-prME, BinJ/DENV2-prME + QA (Desert King), DENV2 VLP (The Native Antigen), DENV2 VLP + QA and non-vaccinated control groups (saline only). Each mouse received 1 μg of BinJ/DENV2-prME or 1 µg of DENV2 VLP, either with or without 3 µg of QA. All mice were immunized three times with 21 days between booster doses. Tail bleeds were taken on day 0, 20, 41 and 51 post first immunization The serum fraction of each sample was recovered by leaving the blood samples to clot overnight at 4 °C, then centrifuging them at 10,000 × *g* for 10 min at 4 °C and stored at −20 °C until further analysis.

### Immunization of AG129 mice with vaccine candidate: HD-MAP study

Female AG129 mice (6–8 weeks old) were divided into six groups of eight mice for immunization by either ID injections or HD-MAPs delivery with BinJ/DENV2-prME or non-vaccinated control groups (saline). Each mouse received 1 μg of purified BinJ/DENV2-prME. Mice (*n* = 16) immunized with BinJ/DENV2-prME were split randomly into two groups (*n* = 8). The first group was challenged after a single vaccination, whereas the second group was given a booster dose 21 days later. Tail bleeds were taken on day 0 and 20 post primary immunization for the single-dose group and on day 41 for the two-dose groups. Sera were collected as stated above.

### Virus challenge

Mice were challenged 10 days (comparative study) and 21 days (HD-MAP study) after the final vaccination. Then, 10^5^ pfu of the mouse-adapted DENV2 D220 strain were injected intraperitoneally into each mouse. Daily weight and tail bleeds were taken for 10 days post infection. Sera were collected as mentioned above, to measure viremia and NS1 viral protein levels. Mice were monitored twice daily for morbidity and terminal bleeds were collected 10 days post infection.

### DENV-specific IgG ELISA

To assess for seroconversion to the vaccine candidates, ELISA plates (Nunc Maxisorb, Thermo Fisher) were coated with 80 ng/well of BinJ/DENV2-prME in PBS overnight at 4 °C. Plates were blocked with blocking buffer for 1 h at room temperature. The vaccinated mouse sera were initially diluted 1 : 100, then 5-fold down a 96-well plate in blocking buffer before adding 50 µL of diluted sera to the ELISA plate and incubated at 37 °C for an hour. Plates were washed four times with PBS-T and were then probed with 50 µL/well of HRP-labelled goat anti-mouse (1 : 1000, Thermo Fisher). TMBW was added after washing the plates for six times with PBS-T and relative absorbance was measured at 450 nm on a plate reader.

### Plaque reduction neutralization tests

Vero cells were seeded at 4 × 10^5^ cells/well in a 96-well plate (Thermo Fisher) and incubated overnight at 37 °C/5% CO_2_. Mouse sera obtained after final vaccination were heat inactivated at 56 °C for 30 min. Sera were diluted 1 : 25 then serially diluted 2-fold down a 96-well plate in serum-free OPTI-MEM. An equal volume of DENV2 ET00 virus stock was added at 75 pfu/well to the sera dilution. The virus–serum mixture was allowed to incubate for 1 h at 37 °C before adding 50 µL of it to the confluent Vero cells. Following a 1 h incubation at 37 °C, the medium was removed and the cells were overlayed with 100 µL of 1.5% carboxymethylcellulose in M199 media (Thermo Fisher) supplemented with 2% FBS. The plates were incubated for 3 days at 37 °C after which the overlay was removed and the cell monolayers washed twice with PBS. Cells were then fixed with 100 µl ice-cold 80% acetone in PBS for 20 min at −20 °C. Fixative was removed and plates were allowed to dry overnight, prior to immunofluorescence staining described below.

### Immunofluorescence staining

Fixed PRNT or plaque assay plates were blocked with 150 µL/well of 0.1% BSA in PBS for 1 h at room temperature. After removing the blocking buffer, 50 µL/well of 1 : 1000 diluted rabbit anti-NS1 polyclonal antibody in blocking buffer was added and incubated at 37 °C for 1 h. Following four washes with PBS-T, 50 µL/well of 1 : 1000 diluted donkey anti-rabbit IR800 (Millennium) antibody was added and the plates incubated for a further 1 h at 37 °C. The plates were washed six times with PBS-T and allowed to dry completely before visualizing and imaging on the Odyssey Clx machine. Plaques were counted and virus titres were determined.

### Quantification of viremia by plaque assays

Vero cells were seeded at 4 × 10^5^ cells/well in a 96-well plate (Thermo Fisher) overnight at 37 °C. On a separate 96-well plate, sera obtained during daily bleeds post infection were first diluted 1 : 10 then serially diluted twofold down the plate in OPTI-MEM media. Media were removed from cells, and 50 µL of serially diluted sera were added to the cells and allowed to incubate at 37 °C for 2 h. After incubation, the serially diluted sera were removed and the cells were overlayed with 1.5% carboxymethylcellulose as described for the PRNTs. Immunofluorescence staining was performed as stated above.

### NS1 quantitative capture ELISA

NS1 capture ELISA was performed as described by Muller et al.^15^. Briefly, plates (Nunc Maxisorb) were coated with 50 µL of 5 µg/mL anti-NS1 monoclonal antibody in bicarbonate buffer overnight at 4 °C. Plates were incubated in blocking buffer (1% sucrose in 1× KPL in PBS-T) for 1 h at room temperature. Mouse sera obtained from the daily bleeds during the 10-day challenge were diluted 1 : 100 and 50 µL of the diluted sera was added to each well in duplicates. Plates were incubated for 1 h at 37 °C and then were washed 4× with PBS-T. Fifty microlitres of detection antibody (anti-NS1-HRP; Panbio) was added into each well and incubated for 1 h at 37 °C. Plates were then washed 6× with PBS-T before adding 50 µL/well of TMBW. Absorbance was read at 450 nm and quantified against a standard curve plotted using samples of known NS1 concentration.

### Statistical analysis

All anaylses were performed using GraphPad Prism version 7.0 (San Diego, CA, USA). One-way analysis of variance was performed for multiple comparison analysis with the α-level set at 0.05 with a Tukey’s post test.

### Reporting summary

Further information on research design is available in the Nature Research Reporting Summary linked to this article.

## Supplementary information

Supplementary Information

Reporting Summary

## Data Availability

The authors declare that all data supporting the findings of this study are available within the paper and its Supplementary Information files.
