# Supplementary material for: A chimeric dengue virus vaccine candidate delivered by high density microarray patches protects against infection in mice
Source: NPJ Vaccines. 2021 May 7;6:66. doi: 10.1038/s41541-021-00328-1 (PMC8105366; doi:10.1038/s41541-021-00328-1)
Supplement: Supplementary file 1 — Supplementary Information [file 41541_2021_328_MOESM1_ESM.pdf]

Supplementary Information

Supplementary Figure 1. Percentage weight loss and morbidity scores following virus challenge in dose optimisation study (with Fig. 3)

Supplementary Figure 2. IFA analysis by confocal microscopy of BinJ/DENV2-prME and DENV2 infected C6/36 and mammalian cell lines

Supplementary Figure 3. Percentage weight loss and morbidity scores following virus challenge in HD-MAP study (with Fig. 6).

Supplementary Figure 4. SDS-PAGE (4 – 12%) analysis of gradient purified BinJ/DENV2-prME alongside BSA standards (with Fig. 1).

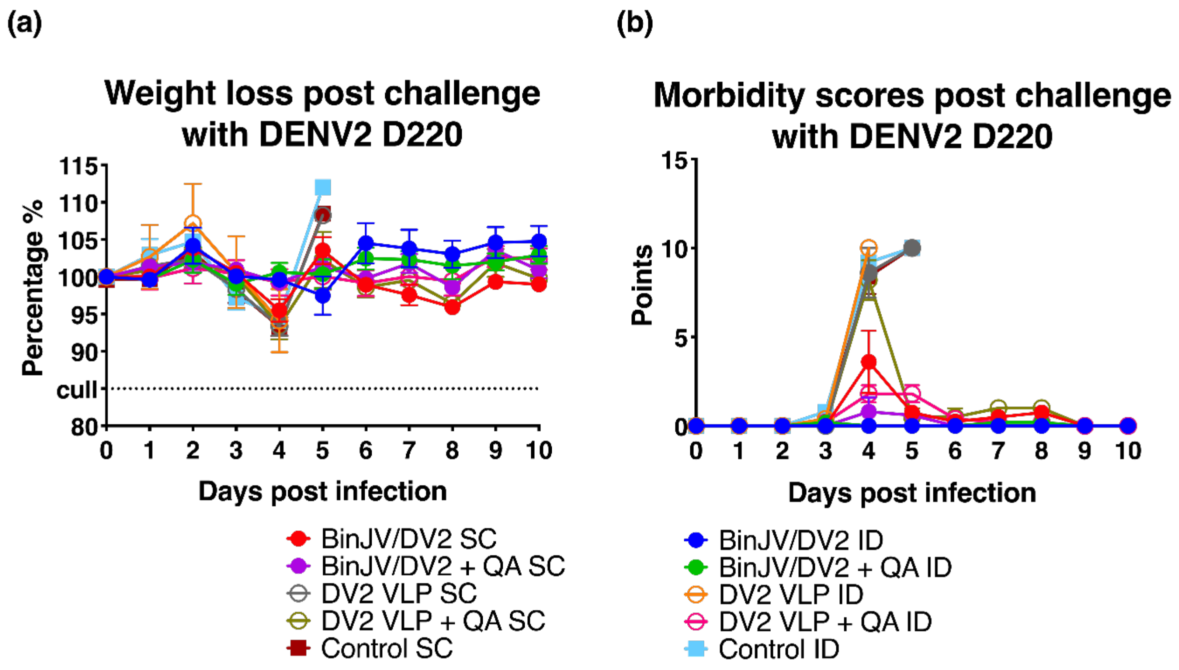

Supplementary Figure 1. Percentage weight loss and morbidity scores following virus challenge in dose optimisation study (with Fig. 3). **(a)** Mean percentage weight loss per group (*n*=5) across 10 days post DENV2 D220 virus challenge. Dotted line represents 15% weight loss. (b) Mean morbidity scores per group (*n*=5) across 10 days post DENV2 D220 virus challenge.

1

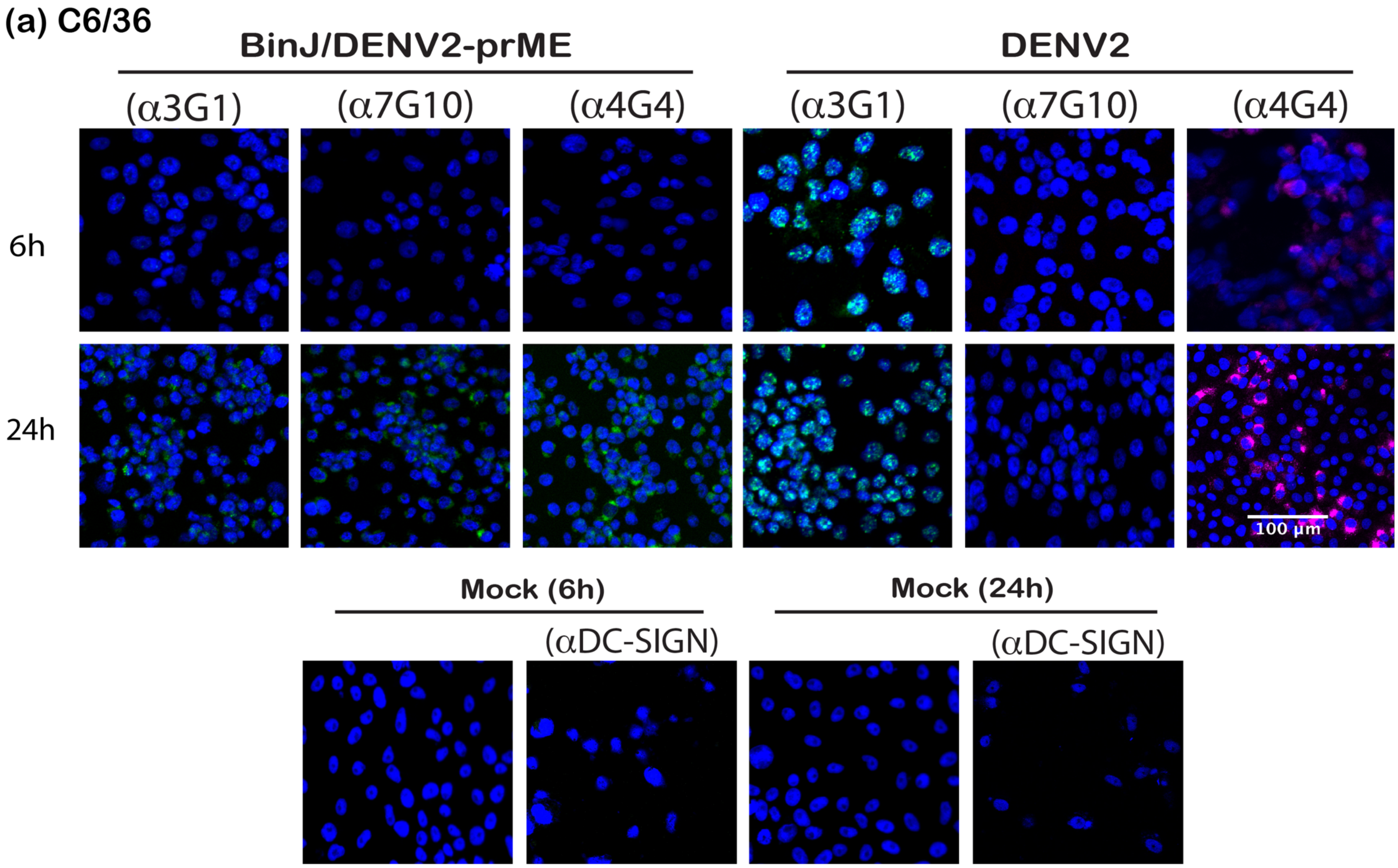

α3G1: dsRNA; α7G10: BinJV specific NS1; α4G4: pan-flavivirus NS1

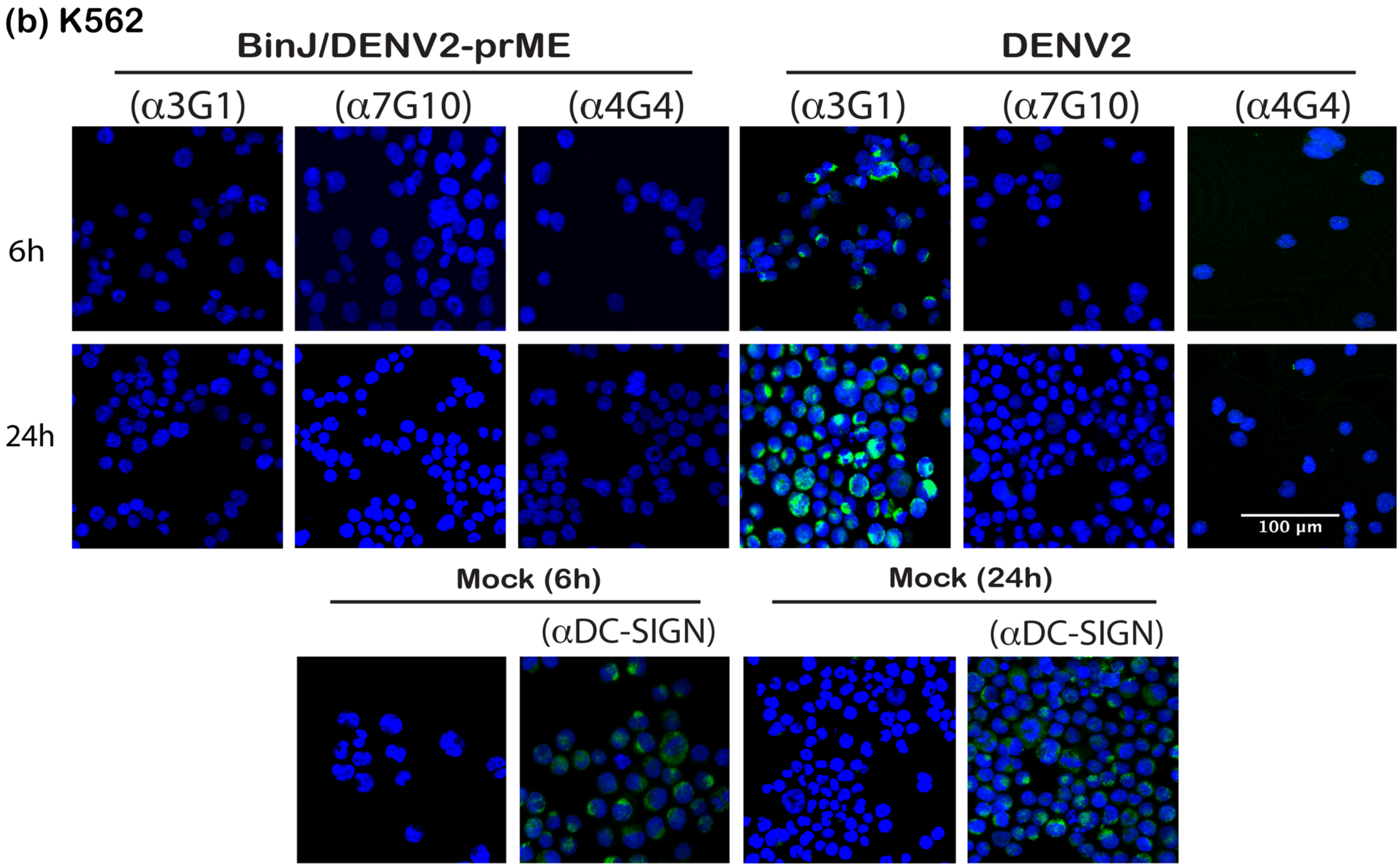

α3G1: dsRNA; α7G10: BinJV specific NS1; α4G4: pan-flavivirus NS1

2

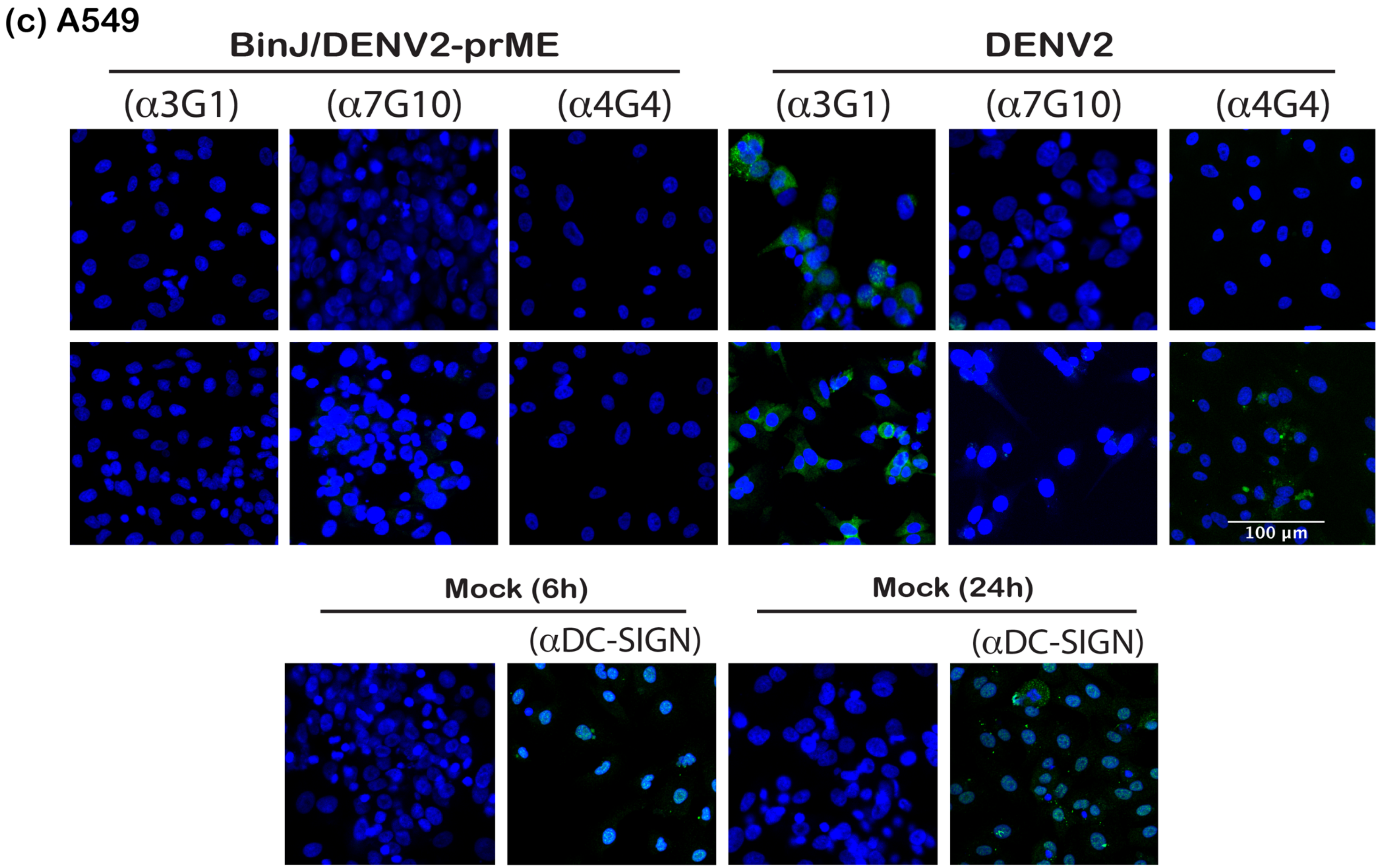

3 α3G1: dsRNA; α7G10: BinJV specific NS1; α4G4: pan-flavivirus NS1

Supplementary Figure 2. IFA analysis by confocal microscopy of BinJ/DENV2-prME and DENV2 infected C6/36 and mammalian cell lines. **(a)** C6/36, **(b)** K562 and **(c)** A549 cell lines were infected with BinJ/DENV2-prME or DENV2 (MOI: 1) and fixed 6 and 24 hours post infection. Viruses were stained with mAb 3G1 (dsRNA; green), 7G10 (BinJV specific NS1; green), 4G4 (pan-flavivirus NS1; green or magenta) and cell nuclei were stained with Hoechst 33342 (blue). Mock infected cells were immunolabelled with anti-DC-SIGN antibodies to confirm the absence/presence of DC-SIGN receptors (green).

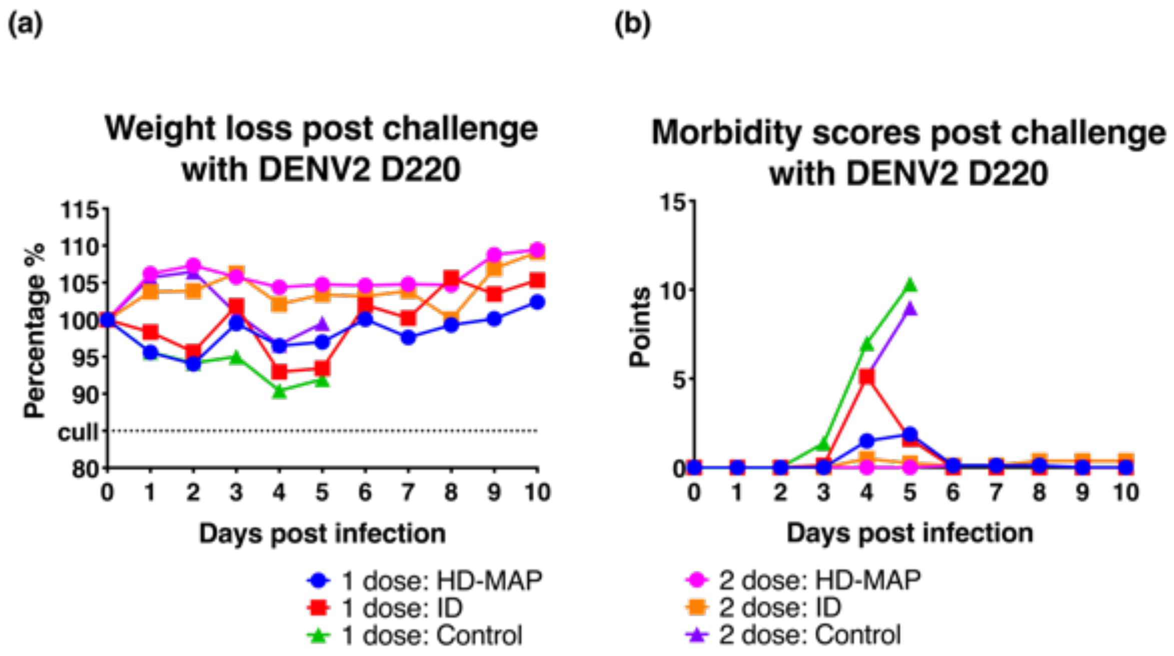

Supplementary Figure 3. Percentage weight loss and morbidity scores following virus challenge in HD-MAP study (with Fig. 6). **(a)** Mean percentage weight loss per group (*n*=8) across 10 days post DENV2 D220 virus challenge. Dotted line represents 15% weight loss. (b) Mean morbidity scores per group (*n*=8) across 10 days post DENV2 D220 virus challenge.

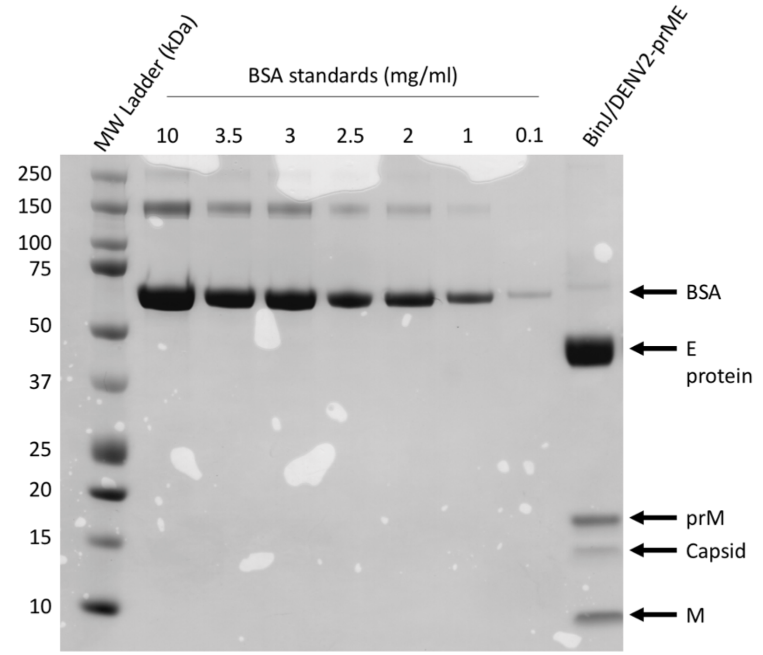

Supplementary Figure 4. SDS-PAGE (4 – 12%) analysis of gradient purified BinJ/DENV2-prME alongside BSA standards (0.1, 1, 2, 2.5, 3, 3.5 and 10 mg/mL). Flavivirus structural proteins (pre-membrane (prM), C , membrane (M) and envelope (E) protein) are indicated.
