# Supplementary material for: A chimeric dengue virus vaccine candidate delivered by high density microarray patches protects against infection in mice
Source: NPJ Vaccines. 2021 May 7;6:66. doi: 10.1038/s41541-021-00328-1 (PMC8105366; doi:10.1038/s41541-021-00328-1)
Supplement: Supplementary file 2 — Reporting Summary [file 41541_2021_328_MOESM2_ESM.pdf]

nature research

Corresponding author(s): Jody Hobson-Peters and David A. Muller

Last updated by author(s): Mar 31, 2021

Reporting Summary

Nature Research wishes to improve the reproducibility of the work that we publish. This form provides structure for consistency and transparency in reporting. For further information on Nature Research policies, see our Editorial Policies and the Editorial Policy Checklist.

Statistics

For all statistical analyses, confirm that the following items are present in the figure legend, table legend, main text, or Methods section.

n/a	Confirmed	
☐	☒	The exact sample size (*n*) for each experimental group/condition, given as a discrete number and unit of measurement
☐	☒	A statement on whether measurements were taken from distinct samples or whether the same sample was measured repeatedly
☐	☒	The statistical test(s) used AND whether they are one- or two-sided<br>*Only common tests should be described solely by name; describe more complex techniques in the Methods section.*
☒	☐	A description of all covariates tested
☐	☒	A description of any assumptions or corrections, such as tests of normality and adjustment for multiple comparisons
☐	☒	A full description of the statistical parameters including central tendency (e.g. means) or other basic estimates (e.g. regression coefficient) AND variation (e.g. standard deviation) or associated estimates of uncertainty (e.g. confidence intervals)
☒	☐	For null hypothesis testing, the test statistic (e.g. *F*, *t*, *r*) with confidence intervals, effect sizes, degrees of freedom and *P* value noted<br>*Give P values as exact values whenever suitable.*
☒	☐	For Bayesian analysis, information on the choice of priors and Markov chain Monte Carlo settings
☒	☐	For hierarchical and complex designs, identification of the appropriate level for tests and full reporting of outcomes
☒	☐	Estimates of effect sizes (e.g. Cohen's *d*, Pearson's *r*), indicating how they were calculated

Our web collection on statistics for biologists contains articles on many of the points above.

Software and code

Policy information about availability of computer code

Data collection	No software was used.
Data analysis	Graphpad Prism was used to analyse all data and its statistics.

For manuscripts utilizing custom algorithms or software that are central to the research but not yet described in published literature, software must be made available to editors and reviewers. We strongly encourage code deposition in a community repository (e.g. GitHub). See the Nature Research guidelines for submitting code & software for further information.

Data

Policy information about availability of data

All manuscripts must include a data availability statement. This statement should provide the following information, where applicable:

- Accession codes, unique identifiers, or web links for publicly available datasets
- A list of figures that have associated raw data
- A description of any restrictions on data availability

The authors declare that all data supporting the findings of this study are available within the paper and its supplementary information files.

Field-specific reporting

Please select the one below that is the best fit for your research. If you are not sure, read the appropriate sections before making your selection.

☒ Life sciences ☐ Behavioural & social sciences ☐ Ecological, evolutionary & environmental sciences

For a reference copy of the document with all sections, see nature.com/documents/nr-reporting-summary-flat.pdf

Life sciences study design

All studies must disclose on these points even when the disclosure is negative.

Sample size	Statistical Justification: The sample size was calculated using G*Power 3.1.5. Using the following parameters: alpha=0.05; power=0.90; number of groups=8; to detect at least 70% difference of the mean with the control group.
Data exclusions	No data was excluded from analysis
Replication	Animal experiments were powered to sufficient n (8).
Randomization	Mice from the breeding colony were randomly assigned to experimental groups.
Blinding	Sera was randomized and blindly tested.

Reporting for specific materials, systems and methods

We require information from authors about some types of materials, experimental systems and methods used in many studies. Here, indicate whether each material, system or method listed is relevant to your study. If you are not sure if a list item applies to your research, read the appropriate section before selecting a response.

Materials & experimental systems

n/a	Involved in the study	
☐	☒	Antibodies
☐	☒	Eukaryotic cell lines
☒	☐	Palaeontology and archaeology
☐	☒	Animals and other organisms
☒	☐	Human research participants
☒	☐	Clinical data
☒	☐	Dual use research of concern

Methods

n/a	Involved in the study	
☒	☐	ChIP-seq
☒	☐	Flow cytometry
☒	☐	MRI-based neuroimaging

Antibodies

Antibodies used	hC8, h513, m4G4, m7G10, m3G1 and polyclonal rabbit NS1: generated in house<br>goat anti-mouse HRP (Thermo-Fisher), donkey anti-rabbit IR800 (Millennium), anti-NS1-HRP (Panbio): obtained from respective companies
Validation	hC8 (Dejnitattisai et al, 2015), h513 (Wong et al, 2018), m4G4 (Clark et al, 2007), m3G1 (O'Brien et al, 2015), m7G10 (Harrison et al, 2020), polyclonal rabbit anti-NS1 was generated by vaccinating a rabbit with S2 expressed DV2 NS1. Polyclonal sera was validated for NS1 binding by western blot and NS1 capture ELISA (Young et al, 2000), goat anti-mouse HRP (Thermo-Fisher), donkey anti-rabbit IR800 (Millennium), anti-NS1-HRP (Panbio).

Eukaryotic cell lines

Policy information about cell lines

Cell line source(s)	Vero, C6/36, A549, K562 (ATCC)
Authentication	N/A
Mycoplasma contamination	Cell lines were confirmed to be mycoplasma free
Commonly misidentified lines (See ICLAC register)	N/A

Animals and other organisms

Policy information about studies involving animals; ARRIVE guidelines recommended for reporting animal research

Laboratory animals	Female AG129 (alpha-, beta-, gamma- receptor knockout) mice (6 – 8 weeks old)
Wild animals	Study did not involve wild animals
Field-collected samples	Study did not involve field collected samples
Ethics oversight	Animal experiments were approved by the University of Queensland animal ethics committee (AEC No: SCMB/AIBN/150/16/NHMRC) and performed in accordance with National Health and Medical Research Council guidelines

Note that full information on the approval of the study protocol must also be provided in the manuscript.
